# End-of-life decisions and opinions: Results of E.L.D.Y.-CA.RE study carried out at Veneto Institute of Oncology, Italy

**DOI:** 10.1177/03008916261437928

**Published:** 2026-06-30

**Authors:** Valter Giantin, Chiara Curreri, Caterina Ugolini, Chiara De Toni, Silvia Stragliotto, Sara Marin, Lavinia Patetta, Marco Maruzzo, Alessandra Feltrin, Sara Lonardi, Antonella Brunello, Vittorina Zagonel

**Affiliations:** 1 Geriatrics Unit, Azienda ULSS 7 Pedemontana, Bassano del Grappa (VI), Italy; 2 Division of Geriatrics, University Hospital of Padua, Padua, Italy; 3 Caterina Ugolini Department FISPPA-Applied Psychology, University of Padua, Padua, Italy; 4 Medical Oncology 1, Veneto Institute of Oncology IOV-IRCCS, Padua, Italy; 5 Medical Oncology 3, Veneto Institute of Oncology IOV-IRCCS, Padua, Italy; 6 Geriatric Unit, Fondazione IRCCS Cà Granda Ospedale Maggiore Policlinico, Milano, Italy; 7 Hospital Psychology, Veneto Institute of Oncology IOV-IRCCS Padua, Italy

**Keywords:** end-of-life decisions, life-sustaining treatment, withhold or withdraw treatments, cancer patients, communication, terminal care, continuous deep sedation

## Abstract

**Background:**

Issues related to end-of-life decisions (ELDs) generate impassioned debates in society. Our study aimed at investigating attitudes in care of cancer patients at the end-of-life (EOL); ascertaining the opinion of healthcare professionals (HCPs), patients, and caregivers.

**Methods:**

An anonymous and self-administered questionnaire was delivered to HCPs (physicians, nurses, psychologists, and social workers), patients and family caregivers at Veneto Institute of Oncology, Italy, and 425 questionnaires were collected (136 HCPs, 171 patients, 118 caregivers).

**Results:**

Withholding life-sustaining treatment (LST) was declared by 11.3% of physicians and nurses and withdrawing by 19.4%, whereas 46.8% declared starting LST, and 48.4% did not discontinue LST. Most HCPs (75.7%) were in favor of patient's right to anticipate EOL, and 86.4% of withholding/withdrawing LST upon patient's request, with 62.4% HCPs agreeing on lethal drug doses to be allowed upon request of the patient. The majority (80.1%) disagreed that “*life is an unavailable asset and there is no right to die*”. For patients and caregivers, in ELDs greater value was given to self-determination first, with family members and trustees’ subsequent involvement. A considerable number of HCPs reported feeling inadequate to communicate bad news.

**Conclusion:**

Despite the limitations imposed by the cross-sectional nature, this study adds information with regard to EOL in oncology setting in Italy. Our study shows that an attitude to prolonging patient's life in ELDs is still prevalent among HCPs in an Italian oncological setting, although there are signs of change. Communicating bad news emerges as an issue, along with HCPs’ awareness that they need more education and training.

## Introduction

Concerns about end-of-life decisions (ELDs) generate impassioned debates in society and continue to divide people.^1,2^ Considerable differences exist among physicians’ attitudes towards taking measures to end lives prematurely, partly subordinated to the national laws.^2-7^ ELDs occur throughout the world, albeit at different frequencies for different actions.^8-12^ The EURELD project was the first study on ELDs based on a review of medical records of a large representative sample of patients who died in six European countries, including Italy.^13^ In this retrospective report, no-treatment decisions were made with frequencies ranging from 14% to 28% in the European countries, and in Italy in 4% of all deaths. Continuous deep sedation was provided in 8.5% of cases in Italy, a percentage that was among the lowest in Europe. Also, in Italy, more than 50% of ELDs were not discussed with either the patient nor with their family.^13^ The second EURELD study, investigating physicians’ clinical attitude on ELDs through a questionnaire, showed Italian physicians had the lowest experience in Europe of foregoing life sustaining treatment (37%, versus 72–86%), and the highest intention of not doing so (24% versus 1– 6%).^2^ Experience with deep sedation until death was also lower in Italy (12%), and hastening death at patient’s explicit request was not performed.^4^ Abstaining from treatment estimated to prolong life for longer than one month was more common in other countries (5%- 10%) compared to Italy (3%) and Sweden (2%).^10^

In the current Italian justice system, life remains an inalienable and legally protected right. After decades-long debate, in 2017 the Italian parliament approved law n. 219 “*Provisions for informed consent and advance directives*” regarding challenging legal and bioethical issues related to ELDs.^14^ The law promotes the person’s autonomy, and a patient-physician relationship shifted to a patient-centered approach; the person’s view, preferences and wills are valued, and the goal of the therapeutic relationship is the pursuit of the patient’s health. This law attests to the patient’s right to withhold or withdraw from life-sustaining treatments. However, real-life practices and opinions of healthcare professionals (HCPs), which directly impact the clinical translation of the new Italian law, are still lacking.^15^ In this context, the role played by nurses in ELDs has not been fully explored.^16,17^ Also, very few studies have compared HCPs’ opinion on ELDs with patients and family caregivers from the same hospital.

Since 2013 Veneto Institute of Oncology (IOV) has been recognized as ESMO Designated Center of Integrated Oncology and Palliative care. A service for palliative care has been active since then, and a procedure for palliative sedation for the management of intolerable suffering in the terminal phase was defined. Such a procedure, which also describes differences between palliative sedation and practices that may shorten life was shared with specific training among HCPs.

Our study aimed to: a) Investigate the practice adopted for end-of-life (EOL) care of cancer patients in a National Comprehensive Cancer Center with particular focus on providing, withdrawing, and withholding aggressive life-sustaining treatments; b) Investigate values and goals of EOL care; c) Ascertain and compare the opinion on artificial nutrition/hydration by HCPs, patients, and caregivers; d) Verify communication on ELDs by HCPs with the patient and/or family.

## Methods

This is a cross-sectional, single center, descriptive study conducted between June and September 2018 at Veneto Institute of Oncology (IOV), Comprehensive Cancer Centre, Padova, Italy.

The study was addressed to cancer patients, HCPs (including physicians, nurses, psychologists, social workers), and family caregivers at the same time.

### Questionnaires

The study, called E.L.D.Y.-CA.RE. (End of Life Decisions studY – CAregivers RElatives), resumed the E.L.D.Y. questionnaire, adapted for the Italian context.^17,18^ In order to mitigate social desirability bias, the questionnaire avoided direct questions as much as possible, and used neutral language. The HCPs’ questionnaire was organized into three parts: the first was aimed only at physicians and nurses and investigated the ELDs they adopted in the most recent experience of caring for patients at the EOL; the second, extended to all HCPs, investigated opinions and attitudes towards EOL. The third and last part aimed at investigating HCPs general characteristics. The adapted questionnaires for patients and caregivers investigated opinions and beliefs about ELDs they had before hospitalization, aimed to explore their opinions on ELDs during hospitalization and communication with HCPs, and collect respondents’ socio-demographic information. The questionnaire responses and associated raw data are available and can be consulted upon request by the authors.

### Sampling and recruitment

Participation in the study was proposed to all HCPs working at IOV. People were invited to participate in the survey through direct contact. The self-administered questionnaire guaranteed complete anonymity by being dropped off by the participants themselves in ballot boxes specifically placed for this purpose within the Institute. Patients provided written informed consent to participate. The study was approved by the Ethics Committee for Clinical Trials (n.9254/2018) of the Veneto Institute of Oncology in Padua, Italy.

### Statistical analysis

This cross-sectional study used a self-report survey methodology. Descriptive analyses were performed to summarize responses to the questionnaires. Comparisons were tested using chi-squared tests or Fisher’s exact tests, as appropriate. For the purpose of statistical analysis, the five response categories for the statements were split into three: (1) agree or strongly agree, (2) neutral and (3) disagree or strongly disagree. Potential differences in professionals' responses by gender, age (<50 years, ⩾50 years), training in palliative care and/or bioethics were assessed. The comparisons were tested using chi-squared tests or Fisher’s exact tests, as appropriate. R Version 4.3.2 was used to perform all statistical analyses. The level of significance was set at 5%.

## Results

Between June and September 2018, 425 questionnaires were collected, accounting for 35.4 % of the questionnaires which were delivered (425/1200). One hundred thirty-six HCPs participated in the survey, of which 38 were physicians, 56 nurses, seven psychologists, 16 social workers and 19 other professionals, constituting 32% of the sample; 171 patients (40.3%) and 118 family caregiver (27.7%) also took part. Socio-demographic characteristics of the participants are summarized in Table 1.

**Table 1.** Participants’ characteristics (n=425).

	Physicians n (%) 38 (28)	Nurses^*^ n (%) 56 (41)	Psychologist n (%) 7 (5)	Social Workers n (%) 16 (12)	Others** n (%) 19 (14)	Patients^§^ n (%) 171	Relatives^§§^ n (%) 118
**Gender**							
Male	14 (37)	6 (11)	1 (14)	3 (19)	6 (32)	89 (56)	25 (22)
Female	24 (63)	48 (89)	6 (86)	13 (81)	13 (68)	71 (44)	87 (78)
**Age (years)**							
< 50	33 (87)	35 (63)	7 (100)	11 (69)	14 (74)	30 (19)	37 (34)
⩾ 50	5 (13)	21 (38)	0 (0)	5 (31)	5 (26)	126 (81)	73 (66)
**Physician specialization**							
Oncologist	28 (74)						
Surgeon	4 (10)						
Radiotherapist	3 (8)						
Anesthetist	1 (3)						
Other	2 (5)						
**Education (patients and relatives)**							
Elementary school						11 (7)	5 (4)
Middle school						40 (25)	25 (22)
High school						76 (47)	53 (47)
Degree						34 (21)	30 (27)

* Nurses missing: 2 for sex. **Technical 4, Pharmacist 3, Studies coordinator 2, Physiotherapist 1, Logopedist 1, Administrative 2, Volunteer 3, Missing 3. ^§^ Patients missing: 11 for sex, 15 for age, 10 for education. ^§§^ Relatives missing: 6 for sex, 8 for age, 5 for education.

### Providing, withdrawing, and withholding aggressive life-sustaining treatments

Thirty-five physicians (92.1%) and 29 nurses (51.8%) claimed they had assisted patients until death during the last year. With regard to the question: “*Did you perform one or more of the following actions, taking into consideration the possibility that this action could prolong life of a patient in the terminal phase*” 46.8% of the sample, of which 18 (64.3%) were nurses and 11 (32.3%) physicians (p= 0.02) declared they “*started life-sustaining treatment*”; 48.4% of the sample, of which 19 (67.9%) were nurses and 11 (32.3%) physicians (p=0.01), declared that they “*did not discontinue life support”* for the same reason. Three percent of physicians, and 43% of nurses (p=0.0001) declared that they had continued unnecessary treatment upon pressing request from patients’ relatives.

To the question: “*Did you perform one or more of the following actions, taking into consideration the possibility that this action could abbreviate the end of the patient's life*”, life support withholding was declared by 11.3% and withdrawal by 19.4%, with no difference between physicians and nurses. Detailed responses are reported in Online Supplementary Material S1. Doctors and nurses reported that in 75% of cases these actions were implemented following patient’s request (in 25% for explicit request, and previously expressed wish in 50% of cases).

Deep sedation had been resorted to in 75% of the deceased patients. Physicians were more likely to share ELDs with a colleague (71.4%), with a family member of the patient (57.1%) and less likely to share with the patient and nurses (14.3% in both cases). Nurses declared they discussed ELDs in 50% of cases with a family caregiver, and only in 25% with another nurse or a physician.

### Values and goals of EOL care according to HCPs

In this multiple answer question, the main aim of care at the EOL was pain relief for 125 (92.6%) HCPs, quality of life for 113 (83.7%), psychological needs for 76 (56.3%), spiritual needs for 49 (36.3%), and cancer-directed treatment for 11 (8.1%). Furthermore 51.1% of HCPs declared attention to family members, and 35.6% also concerns for the partner. The agreement by groups of HCPs was very high (Figure 1a). With regard to their main focus while assisting terminally ill patients, this was respect for patient's dignity for 122 (91%) of those interviewed, respect for patient's will for 99 (73.9%), improving quality of life for 98 (73.1%), respect for life for 41 (30.6%), justness when considering all patients for 10 (7.5%).

**Figure 1.**
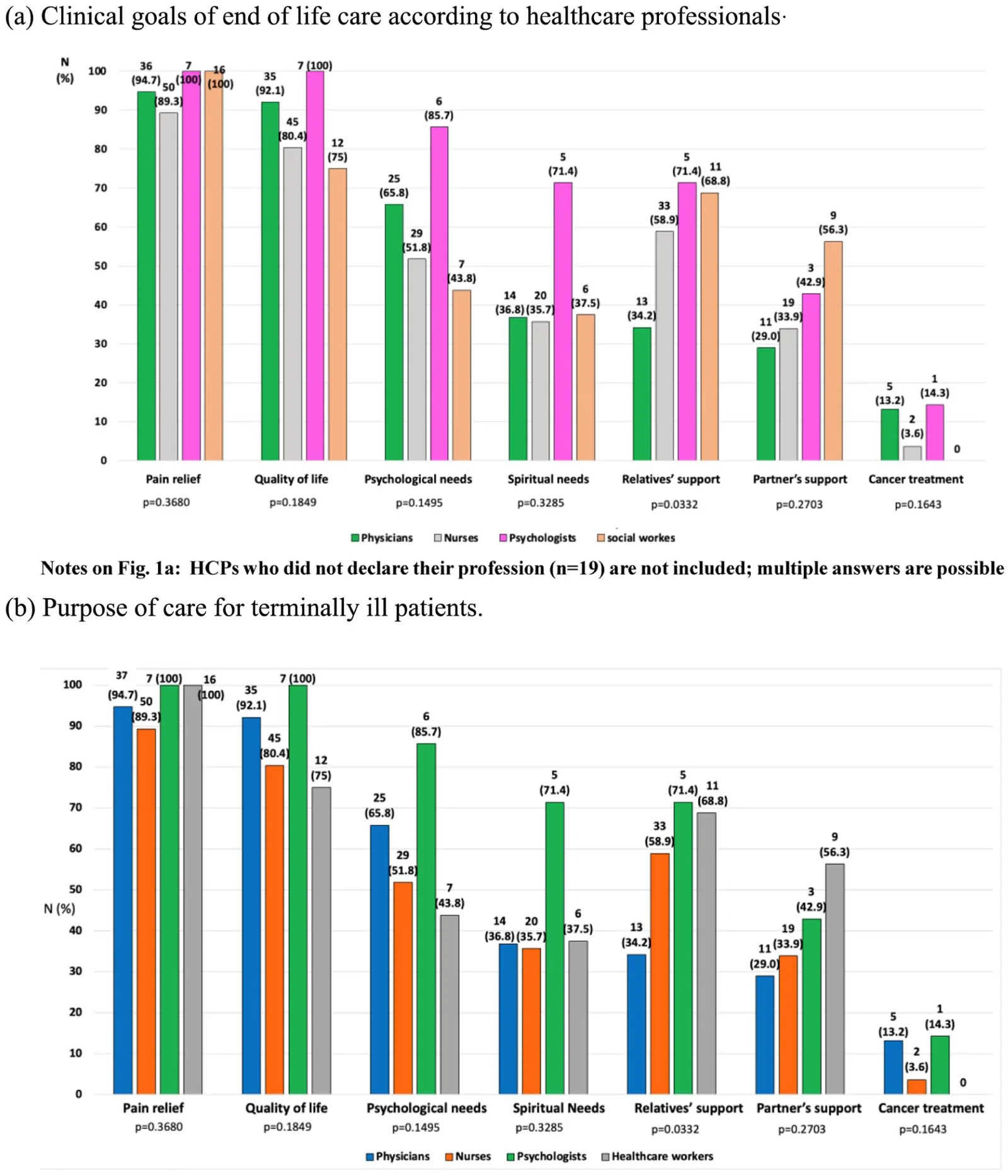
(a) Clinical goals of end of life care according to healthcare professionals: HCPs who did not declare their profession (n=19) are not included; multiple answers are possible. (b) Purpose of care for terminally ill patients.

No significant differences in responses were found between the different groups of healthcare professionals (Figure 1b).

Table 2 details the results of the section of the questionnaire exploring HCPs’ views on ELDs. Responses above 50% of the sample are highlighted in the table. No statistically significant differences were observed in the responses within the subgroups of HCPs.

**Table 2.** Opinions of HCPs on end-of-life decisions (ELDs).

Opinions of HCPs on ELDs	Agree N (%)	Neutral N (%)	Disagree N (%)
The availability of palliative care prevents almost all requests for euthanasia or assisted suicide.	**72 (54.6)**	20 (15.5)	40 (30.3)
Life is an unavailable good and there is no “right to die”.	12 (9.2)	14 (10.7)	**105 (80)**
A patient’s request to withhold or discontinue life-sustaining treatments should be honored.	**115 (86.5)**	12 (9.0)	6 (4.5)
In the case of an incompetent patient, relatives should be allowed to decide on life-sustaining treatments.	**72 (54.1)**	37 (27.8)	24 (28.0)
Every person should have the right to decide to bring forward the end of her life.	**100 (75.7)**	16 (12.1)	16 (12.1)
Each person should be allowed to appoint a trustee to make ELDs on his behalf in case of incompetence.	**121 (90.9)**	6 (4.5)	6 (4.6)
Allowing the use of drugs in lethal doses on the explicit request of the patient will gradually lead to an increase in such use, even without a request from the patient.	31 (23.3)	29 (21.8)	**73 (54.9)**
The use of drugs in lethal doses at the explicit request of the patient is acceptable in patients with intolerable suffering at the end-of-life.	**83 (62.4)**	14 (10.5)	36 (27.1)
The administration of drugs in lethal doses should be permitted in the case of patients at the end-of-life with intolerable suffering who are unable to make decisions.	39 (29.8)	25 (19.1)	**67 (51.5)**
Clear wishes on withholding or withdrawing life-sustaining treatment of an incompetent patient as expressed in an advance directive must always be respected, even if this could hasten the end of the patient’s life.	**78 (59.1)**	30 (22.7)	24 (18.2)
An interdisciplinary approach is useful in ELDs. The involvement of family members, if available, is essential for ELDs.	**110 (82.7)** **87(65.4)**	14 (10.5) 25(18.8)	9 (6.7) 21(15.8)

Bold indicates responses above 50%.

### EOL procedures: Comparing opinions by HCPs, patients and family caregivers

Overall, adequate measures to be guaranteed were artificial hydration for 118 (87.4%) of the interviewed HCPs and artificial nutrition for 45 (33.3%), with a significant difference between the professionals (psychologists and social workers were more in favor compared to physicians and nurses p=0.05). Seventy-four HCPs (54.8%) were also in favor of assisted ventilation, and 23 (17%) of dialysis, with a significant difference among operators (p= 0.02). Artificial hydration was to be always guaranteed for the vast majority of physicians and nurses, being considered as routine care. With regard to artificial nutrition, however, unlike nurses, many physicians considered it as medical treatment to be administered only under certain circumstances (16 out of 38 physicians vs. 14 out of 56 nurses, p=0.04).

Patients and caregivers were asked to answer the question: *“What is your opinion on the decision to administer artificial hydration and/or nutrition at the end of life?”*

Quite consistent answers were obtained from patients and relatives. Sixty-four (39%) patients and 42 (35.9%) caregivers believed that “*It should always be the patient or their fiduciary alone who decides*”; 28 (17.1%) and 21 (18%) respectively stated “*I rely totally on the physician providing care*”, and 24 (14.6%) and 16 (13.7%) respectively “*I believe it should be the patient's family members or caregivers who decide”.* Furthermore, 36 (22%) patients and 33 (28.2%) caregivers declared that “*hydration and nutrition should always be implemented because life should always be safeguarded”.*

A significant difference was found in opinions on hydration and artificial nutrition between physicians and nurses compared to patients and caregivers. Although 50.9% of patients and 63.4% of caregivers favored the administration of both hydration and artificial nutrition at the EOL, more than 30% of patients and 13.4% of caregivers did not consider them necessary (Figure 2).

**Figure 2.**
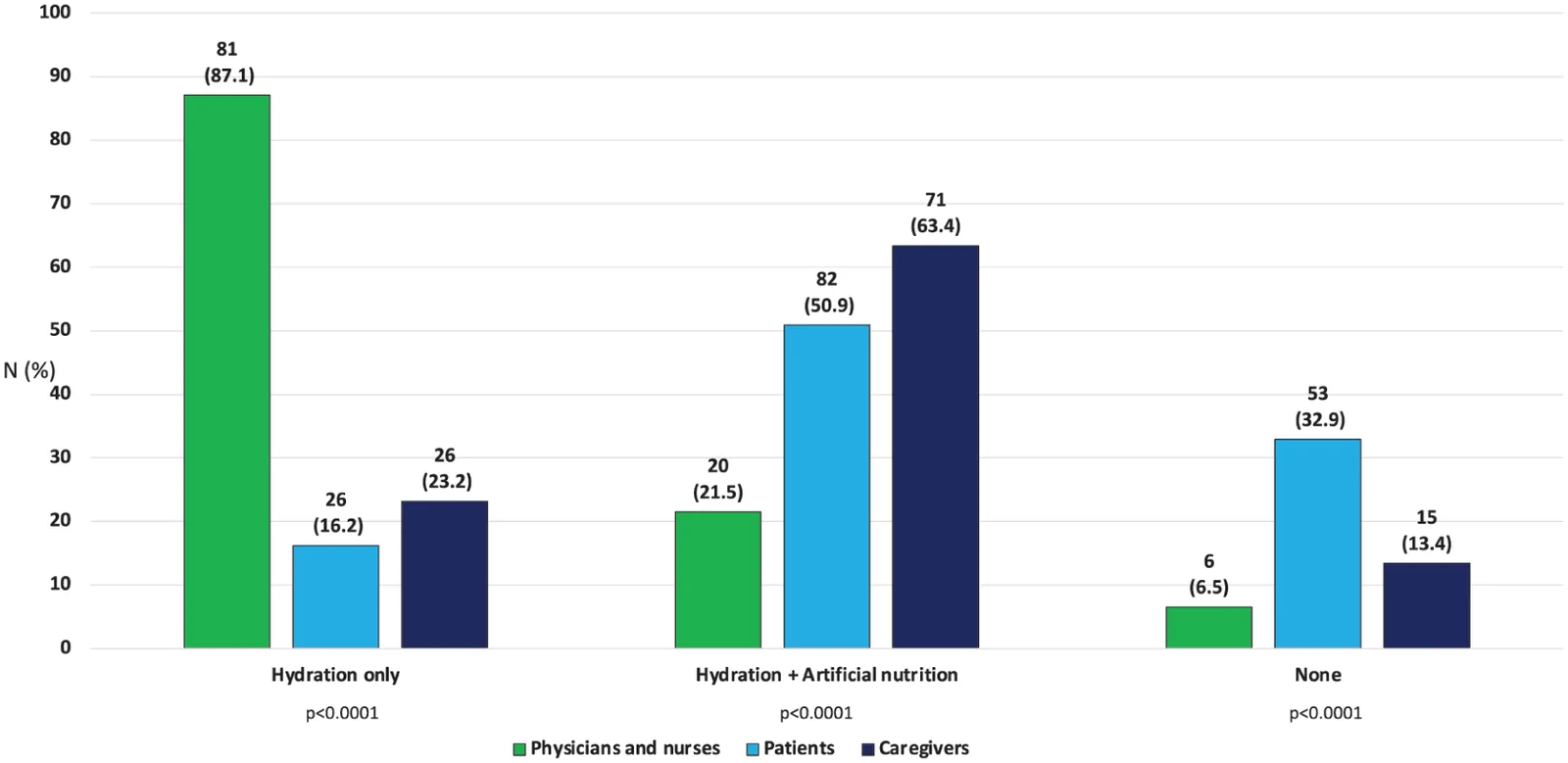
Hydration and artificial nutrition at the end of life: Opinions of physicians and nurses compared to patients and caregivers.

With regard to palliative care, 60.7% of patients and 84% of caregivers acknowledged this was always guaranteed at the EOL, compared to 92.5% of HCPs (p<0.0001).

Patients and caregivers were asked *"If there were no written Advance Directives (ADs), how would you like decisions regarding your EOL to be made?”* and, as reported in Figure 3a, in their opinion family members should be consulted in the decision-making process (52.5% of patients and 63% of caregivers), counting on having verbally expressed ADs (46% and 39.6%, respectively). For 24.6% of patients and 39.5% of caregivers, their will for ELD should be inferred on the basis of previous verbal statements. In addition, 23.7% and 30.9% respectively wished for decisions to be collectively discussed with the entire medical team, and based on the shared care planning (SCP) with the physician (19.9% of patients and 16.5% of caregivers). The appointed trustee should be the one making the decision for 13.6% of patients and 17.3% of caregivers, whereas the decision should be up to the entrusted physician for 5.9% and 1.2%, respectively. For 3.4% of patients and 9.9% of caregivers other staff (e.g., psychologist, social worker, spiritual assistant) should take part in the decision-making process, including nursing staff (1.7% and 2.5%, respectively). Concordance between patients and caregivers to the answers to these questions was very high.

**Figure 3.**
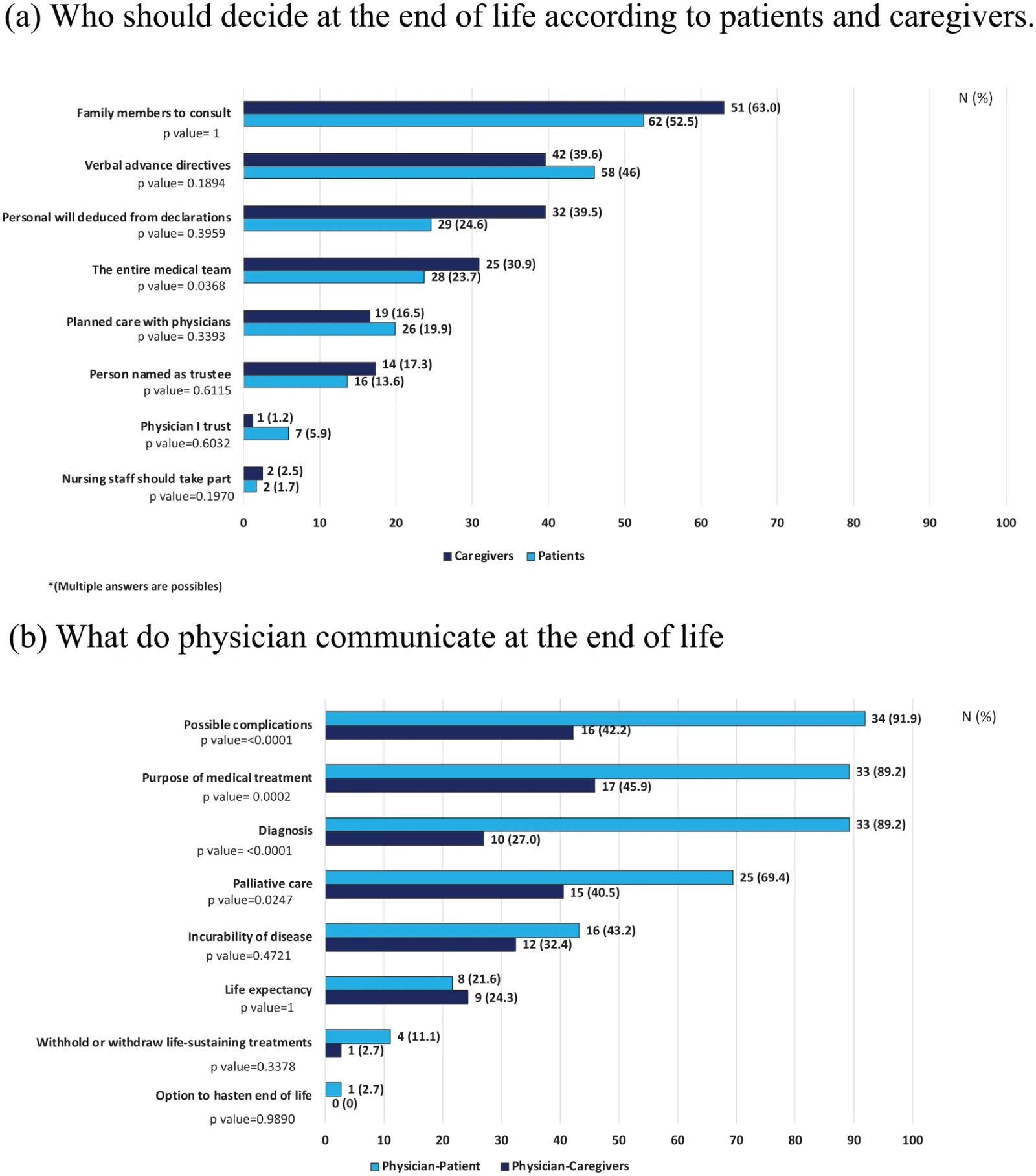
(a) Who should decide at the end of life according to patients and caregivers; (b) What do physician communicate at the end of life.

### ELD and communication between HCPs and patient and/or family member

With regard to the question “*In your clinical practice faced with the possibility of communicating dismal diagnosis, how do you feel?*”, the answer was “Inadequate/insufficient” for 13 physicians (34.2%) and 30 nurses (58.8 %). Regarding the question "*When assisting a patient in the terminal phase of an illness, indicate what topics you always discuss and with whom (patient or caregiver)*”, although results showed propensity to discuss first of all with patients, the content of discussion was mostly not focused on patient’s illness phase. In fact, only 43.2% of physicians stated they always discuss the incurability of the disease with the patient, 21.6% life expectancy to the patient, 11.1% discontinuation of life-sustaining treatments, and 2.8% options to hasten EOL (Figure 3b).

Questionnaires delivered to patients showed that only 7.3% of patients had disclosed ADs, 11.1% had appointed a fiduciary, and 19.9% had discussed SCP, as recommended by Italian law. Consistently with physicians' and nurses' statements, 90% of patients in the terminally ill phase were cared for by the palliative care team, and 75% received continuous deep sedation.

Among interviewed HCPs, no difference in questionnaire responses was found in relation to age, gender, and training in palliative medicine or bioethics (data not shown).

## Discussion

ELDs remain an open ethical and medical issue for patients, HCPs, and family caregivers. In pluralistic societies, with the increasing attention and demand for palliative care, new laws are being implemented to improve EOL care.^7,14^ This survey crosswise collected the opinion of HCPs, patients and relatives on the EOL of cancer patients at the same institution.

Among the major findings of our study is the rate of agreement among HCPs on the more relevant clinical goals, symptoms relief and quality of life in particular, to be pursued at the EOL, mainly aimed at respecting the dignity of the patient and their wishes. Palliative care is widely used. Continuous deep sedation, one the options to reduce intolerable suffering, has been implemented by most respondents in routine EOL care. Despite the limitations imposed by a cross-sectional study, this procedure seems to be appreciably increasing compared with other Italian experiences conducted in the past and in other settings,^17,19-21^ and are similar to other European case histories of cancer patients.^7,12^ In addition, in terms of ethical issues, our HCPs sample demonstrated greater awareness than HCPs of a comparable cancer center in Italy.^22^

Only 31% of HCPs declare respect for patient's life *tout-court* as the aim to be pursued at the EOL. Such data are in line with the low percentage of HCPs who claim that life is an “unavailable good”, while a majority of those interviewed are in favor of patient's right to bring forward the end of their life, and 86.4% are in favor of withholding or withdrawing life-sustaining treatments at the patient's request. Furthermore, 62.4% of interviewed physicians also believe that the use of drugs at lethal doses should be allowed upon request of the patient. These results seem to reflect greater attention to patient's wishes compared to the 2008 survey conducted among Italian oncologists, in which only 39% of respondents were in favor of thorough application of patients’ ADs.^23^

These results indicate first of all a prevailing position in favor of respect of autonomy (*pro-choice)*, rather than observance for life at all costs (*pro-life*). The same principle also emerges with patients and caregivers, who show substantial agreement with regard to the issue of who should make ELDs. Greater value is in fact given by patients to self-determination first in all its expressions (such as written ADs, verbal ADs, wishes inferred from declarations, SCP), and a second place the involvement of family members, and the trustee. The tendency to continue active treatment during the final few months of their patients’ lives is still widespread in Italy, as a recent survey conducted in Liguria pointed out.^24^

On these premises, the predominant attitude of prolonging life in ELDs declared by physicians and nurses, particularly with their low propensity to share these choices with the patients, might appear conflicting. In our survey, indeed, in terminally ill patients cared for in the past years, decisions to anticipate EOL were much less frequent than decisions to keep the patient alive despite unfavorable prognosis. Interestingly, notwithstanding the limitations of a comparison between studies over time, our data show an increase in withdrawing or withholding life-sustaining treatment compared to the rates reported in a previous European survey.^2^ Of particular interest is the comparison of some responses from the two surveys (EURELD study and this study) using the same questionnaire (Table 3). Although the comparison is quite arbitrary, Italian physicians’ opinions seem to have changed over time and show greater consideration for individual freedom. The only countertrend is in regard to the compliance to ADs previously disclosed by the person, in case of a non-competent patient: only half of the physicians who participated in our survey agree. This could be related to the awareness of physicians working in oncology, of how many cancer patients change their minds about their EOL after a cancer diagnosis. Another explanation may be related to the many barriers that discourage the composition of ADs in the oncology setting.^25^

**Table 3.** Degree of agreement on statements on ELDs among Italian physicians: EURELD study and present study.

Degree of agreement on statements on ELDs among Italian physicians	EURELD survey* %	Present survey^ %
Every person should have the right to decide to bring forward the end of her life.	37.0	65.8
Each person should be allowed to appoint a trustee to make ELDs on his or her behalf in the event of incompetence.	48.0	97.4
Physicians should comply with a patient’s request to withhold or withdraw life-sustaining treatment (LST).	62.0	92.1
If a patient is incompetent, relatives should be allowed to decide whether or not to withhold or withdraw LST.	33.0	44.1
The use of drugs in lethal doses at the explicit request of the patient is acceptable in terminal patients with intolerable suffering.	36.0	44.7
Allowing the use of drugs in lethal doses on the explicit request of the patient will gradually lead to an increase in such use, even without a request from the patient.	46.0	26.3
If a terminally ill patient is suffering unbearably and is not capable of making decisions, the physician should be allowed to administer drugs in lethal doses.	26.0	18.9
Clear wishes on withholding or withdrawing LST of an incompetent patient as expressed in an advance directive must always be respected, even if this could hasten the end of the patient’s life.	52.0	50.0
The availability of palliative care prevents almost all requests for euthanasia or assisted suicide.	76.0	68.4
Allowing the use of drugs in lethal doses at the explicit request of the patient will harm the relationship between patients and physicians.	27.0	18.4

* Including only Italian physicians; ^Including only physicians.

In our survey, nurses reported making ELDs more often than physicians (68% versus 44%, respectively). Consensus within the whole care team was the most common way to make ELDs, especially for physicians, and this finding is consistent with results from a previous Italian survey in the anesthesiology^26^ as well as in the geriatric setting.^17^ One major concern raised by our study is that even when physicians and nurses are asked for their opinions, the discussion about prognosis and incurability of the disease seldom occurs unless prompted by the patient, although it is well known that sharing such information can reduce aggressive treatments and provide EOL care more concordant with patient’s preference.^27^ Becoming aware of their limited prognosis may make patients with advanced cancer more receptive to start EOL discussions.^28^ In the EURELD study, forgoing treatment was discussed with the patient or relatives in more than 50% of cases.^10^ Patients and relatives are generally involved in decision-making in countries in which there is a high frequency of such decisions.^3^ The Royal College of Physicians in the UK stated that only 8% of people with cancer who reported thoughts and feelings about their death had shared such considerations with their healthcare team, but only 19% of these conversations were initiated by the HCPs.^29^ A recent survey conducted in Austria showed that physicians discussed the possible consequence of hastened death by withholding a treatment with the patient in 47.2% of cases; in patients dying from cancer, treatment was discontinued in 55.3% of cases.^12^ EOL discussions and ADs were reported by 73% of HCPs in a French study.^7^

The role of nurses in ELDs had already been detected earlier in different countries and settings.^16,17,30^ Nurses are in general positive about their own potential involvement in ELDs.^16^ Although physicians are formally responsible for ELDs, nurses are likely to be involved in the decision-making process. Most nurses think that patients would rather talk to them about ELDs than to physicians,^31^ and focus on their perception of patients’ expectations, severe symptoms and emotional status.^7^ Nurses and physicians have a shared goal, that is to help patients and families through the process of ELDs making. Evidence in the intensive care unit setting shows that a united front of physicians and nurses, stemming from shared decision making that produces clear, consistent messages for families is able to improve the decision-making process.^31^ In a recent Belgian survey, palliative care physicians stress the importance of participation of all stakeholders in the decision-making process to prevent inadequate decisions being made at EOL.^32^

In our study, hydration and artificial nutrition being considered as a treatment rather than care remains controversial for physicians and nurses, with significant differences. These results are consistent with findings from a French experience in the oncology setting.^7^

Our study highlights the demand for specific training in communication which is advocated by physicians and nurses, many of whom reported feeling inadequate to deliver bad news. Communication in the frame of EOL and ELDs is critically important for providing quality care as people approach death.^33^ For cancer patients with a life-limiting illness, conversations about the likelihood of death from their disease should be sensitively offered throughout the disease course. Healthcare professionals need to maintain flexibility in clinical practice and modify the conversations on a case-to-case basis.^34^ From an organizational perspective, early palliative care consultation is the most appropriate setting to communicate with the patient, informing on the evolution of the disease, making them aware of the prognosis, and progressively involve them in ELDs.^35,36^ In fact, patients who received palliative care before their last month of life were nearly two times less likely to experience potentially inappropriate care in the last month of life.^37^ Awareness of actual health status supports the establishment of ADs, appointment of a fiduciary, and/or SCP, all of which were found to be marginal in our survey. As previously reported, patients who had entrusted ADs received EOL care that was strongly adherent with their preferences.^38^ Families need unambiguous communication and honest information to be able to take part in the decision-making process.^39^ Such conversations should be a core professional duty of the healthcare team. A recent Italian survey undertaken in patients with advanced cancer provided evidence on the importance of effective communication strategies between physicians and patients/caregivers regarding illness, understanding, realistic expectations, and planning for the future.^40^ This strategy can disclose patients’ preferences about therapy and outcome, ensuring the possibility to solve doubts or questions regarding EOL treatment options. In this context, communication around death should be viewed as one of the central topics of physician-patient conversation, in line with patients’ and relatives’ readiness and preferences.

Our study has some limitations. First, the results are limited, albeit homogeneous, to a moderate-sized sample of HCPs, cancer patients and family caregivers from a single Institution in Italy, which may not reflect the cultural change throughout Italy.

Despite the measures implemented in the drafting and collection of questionnaires to avoid social desirability bias, we cannot rule out that some favorable responses from HCPs, given the ethically sensitive nature of the topics, may have been influenced by what is considered best practice rather than their true, honest opinions.

Moreover, comparison with previous studies is challenging and sometimes arbitrary because of the heterogeneity of the sample under study, which is often not limited to cancer patients, as well as different countries’ laws that significantly influence behavior in ELDs.

Finally, the study was penalized by the Covid-19 pandemic, which did not allow the data to be processed in a more adequate timeframe.

## Conclusion

This study adds information on the evolving opinion on the EOL in an oncology setting in Italy. Our survey suggest cancer patients, HCPs and family caregivers are in favor of a *pro-choice* position, despite the law in Italy being in favor of life as an unavailable good. Although comparisons with previous surveys remain complex and arbitrary, an attitude of valuing autonomy first appears to be implemented in the Italian oncology setting.

In ELDs, an attitude towards prolonging patient's life, rather than withholding or withdrawing life-sustaining treatments, still prevails, partly justified by pressure from family members. Decision-making at the EOL is only sometimes in line with patients’ wishes, partly because despite patients claims ADs are planned by a minority of patients, including designation of a trustee.

Further studies are needed to confirm such a change in Italian society, and how much this may be affected by the recent law.

Difficulty in communicating bad news emerges from physicians and nurses, along with their awareness of needing more education and training. Engaging in clear and empathetic communication about ELDs could help bridge the gap between desired and experienced EOL for patients and their relatives.

## Supplemental Material

sj-docx-1-tmj-10.1177_03008916261437928 – Supplemental material for End-of-life decisions and opinions: Results of E.L.D.Y.-CA.RE study carried out at Veneto Institute of Oncology, Italy Supplemental material, sj-docx-1-tmj-10.1177_03008916261437928 for End-of-life decisions and opinions: Results of E.L.D.Y.-CA.RE study carried out at Veneto Institute of Oncology, Italy by Valter Giantin, Chiara Curreri, Caterina Ugolini, Chiara De Toni, Silvia Stragliotto, Sara Marin, Lavinia Patetta, Marco Maruzzo, Alessandra Feltrin, Sara Lonardi, Antonella Brunello and Vittorina Zagonel in Tumori Journal

## Data Availability

Questionnaires and data presented in this study are available on request from the corresponding author.
