# Supplementary material for: End-of-life decisions and opinions: Results of E.L.D.Y.-CA.RE study carried out at Veneto Institute of Oncology, Italy
Source: Tumori. 2026 Jun 30;112(5):423–33. doi: 10.1177/03008916261437928 (PMC13687598; doi:10.1177/03008916261437928)
Supplement: Supplementary file 1 [file sj-docx-1-tmj-10.1177_03008916261437928.docx]

**Figure S1: End-of-life decisions**
